# Network-augmented compartmental models to track asymptomatic disease spread

**DOI:** 10.1093/bioadv/vbad082

**Published:** 2023-07-03

**Authors:** Devavrat Vivek Dabke, Kritkorn Karntikoon, Chaitanya Aluru, Mona Singh, Bernard Chazelle

**Affiliations:** The Program in Applied and Computational Mathematics, Princeton University, Princeton, NJ 08544, USA; Department of Computer Science, Princeton University, Princeton, NJ 08544, USA; Department of Computer Science, Princeton University, Princeton, NJ 08544, USA; Department of Computer Science, Princeton University, Princeton, NJ 08544, USA; Department of Computer Science, Princeton University, Princeton, NJ 08544, USA

## Abstract

**Summary:**

A major challenge in understanding the spread of certain newly emerging viruses is the presence of asymptomatic cases. Their prevalence is hard to measure in the absence of testing tools, and yet the information is critical for tracking disease spread and shaping public health policies. Here, we introduce a framework that combines classic compartmental models with travel networks and we use it to estimate asymptomatic rates. Our platform, traSIR (“tracer”), is an augmented susceptible-infectious-recovered (SIR) model that incorporates multiple locations and the flow of people between them; it has a compartment model for each location and estimates of commuting traffic between compartments. TraSIR models both asymptomatic and symptomatic infections, as well as the dampening effect symptomatic infections have on traffic between locations. We derive analytical formulae to express the asymptomatic rate as a function of other key model parameters. Next, we use simulations to show that empirical data fitting yields excellent agreement with actual asymptomatic rates using only information about the number of symptomatic infections over time and compartments. Finally, we apply our model to COVID-19 data consisting of reported daily infections in the New York metropolitan area and estimate asymptomatic rates of COVID-19 to be ∼34%, which is within the 30–40% interval derived from widespread testing. Overall, our work demonstrates that traSIR is a powerful approach to express viral propagation dynamics over geographical networks and estimate key parameters relevant to virus transmission.

**Availability and implementation:**

No public repository.

## 1 Introduction

At the outset of the COVID-19 pandemic, the prevalence of asymptomatic cases among infections was estimated to lie anywhere between 17% and 81% (Nogrady, 2020). Given the importance of this parameter for early health policy decisions (Nishiura *et al.*, 2020), such a high level of uncertainty was a major roadblock. With testing now widely available, this issue has largely dissipated, with estimates of asymptomatic rates between ∼30% and ∼40% (Ma *et al.*, 2021; Shang *et al.*, 2022). To prevent such difficulties in future epidemics, it would be highly beneficial to have computational tools for estimating the asymptomatic rate of infected individuals right at the beginning of an epidemic.

Infectious disease spread is classically modeled using compartmental models. The population is assigned to distinct compartments (e.g. the susceptible, infectious and recovered compartments in the widely studied SIR model) (Kermack and McKendrick, 1927), with rates at which individuals move from one compartment to another. When applying these compartment-based epidemiological models, it is impossible to predict the true prevalence of a virus early on in a pandemic without widespread random testing: indeed, even a tiny fraction of a population showing symptoms for the disease is compatible with a widespread infection. To estimate via computational modeling the fraction of infectious individuals that are asymptomatic, or the *asymptomatic rate* ρ, requires additional information. Here, we show that considering information about how a virus spreads in a spatial manner—not just between compartments at a single location—can be leveraged to estimate ρ. The intuition is that, while individuals travel between locations and this contributes to viral spread, individuals who feel sick (i.e. are symptomatic) tend to curb travel, which in turn yields a distinguishing observable between symptomatic and asymptomatic carriers.

In this article, we introduce traSIR (pronounced “tracer”), a network traffic-based SIR model, which combines the classic SIR compartmental model with network modeling. In traSIR, we have a network where each node is a location (e.g. a county or ZIP Code), each location is associated with a compartmental model and edges in the network represent frequent travel between the locations (e.g. commuting). TraSIR additionally models asymptomatic and symptomatic infections, together with a dampening effect on viral spread for symptomatic infections. Our primary contribution is to demonstrate the utility of traSIR in estimating the asymptomatic rate of an infectious disease using only knowledge about symptomatic infections across geographic locations, as well as information about typical travel between locations.

We begin with theoretical results relating the asymptomatic rate of infection to other key parameters of the model (e.g. infection and recovery rates). Since these key parameters are not known *a priori* and must be estimated from the data, we next assess how well parameters of a traSIR model can be estimated using only knowledge about symptomatic infections. In particular, we simulate disease spread using traSIR, and then perform empirical parameter estimation using the number of symptomatic infections over time across locations to estimate the asymptomatic rate ρ. Across a wide range of parameters, we find excellent agreement between the actual and estimated ρ values. Finally, we analyze the number of reported COVID-19 infections across the New York metropolitan area during the first wave, from March 1, 2020 to September 17, 2020.

The method behind traSIR seeks to combine topological flow information with diagnostic data and behavioral variations. It makes use of a number of observable nonlinearities: (i) in the absence of public health measures, a multiplicative decrease in the symptomatic rate causes a forward time-shift in the infection curve relative to its measurable baseline; (ii) detection of carriers grow superlinearly in the number of symptomatic cases; (iii) the number of newly symptomatic cases is largely determined by the asymptomatic neighbors in the network and (iv) asymptomatic carriers have a different transmissibility rate (Li *et al.*, 2020). Our platform, traSIR, is the first of its kind to integrate county-level data with a commuter network on a large scale to recover critical epidemiological characteristics directly from network dynamics, in particular the asymptomatic rate.

### 1.1 Further background

Standard epidemiological models have previously been extended to account for disease spread across space, but the medium has typically been assumed to be homogeneous (where the population is treated as one large group, as opposed to interacting subpopulations) (Brauer *et al.*, 2008; Eletreby *et al.*, 2020), leading to a diffusive process. Typically, the speed of a wave across the population grows in proportion to the square root of the reproduction number and the diffusion coefficient. Epidemics have also previously been studied in random graphs and scale-free networks (Ajelli *et al.*, 2010; Barrat *et al.*, 2008). Previous work has also considered the correlation of viral spread with changing commuting patterns as well as signals from social media or search engines (Byambasuren, 2020; Li *et al.*, 2020; Poletti *et al.*, 2021; Subramanian and Pascual, 2021; Sun *et al.*, 2021; Zhan *et al.*, 2018); other approaches have integrated network effects into compartmental models (Ameri and Cooper, 2019; Barabási, 2013; Ding *et al.*, 2021; Dottori and Fabricius, 2015; Liu *et al.*, 2018). However, they lack any symmetry-breaking mechanism for distinguishing between symptomatic and asymptomatic carriers. This is precisely what traSIR offers.

Even agent-based modeling does not directly resolve this issue. While agent-based modeling certainly provides enhanced resolution and a distribution of outcomes (certain aspects of which we leverage by decomposing our model to the county level), agent-based modeling has not been able to properly estimate asymptomatic spread (Kerr *et al.*, 2021). One significant downside of agent-based modeling is that it is also computationally expensive, even when leveraging vectorized features to control spread mechanisms (Dabke and Arroyo, 2016).

### 1.2 Symmetry-breaking and asymptomatic spread

In order to properly estimate the asymptomatic rate, we need to be able to distinguish between symptomatic and asymptomatic spread. The model we present in this article allows us to distinguish between these two types of spread via different interaction patterns: we can have varying commuting and travel patterns among asymptomatic and symptomatic populations.

Our results show that under simple assumptions or static parameters, we can still distinguish the spread within these two populations. This implies that having some symmetry-breaking is paramount and while we have ample room for refinement, mere existence of symmetry-breaking is sufficient for estimating the asymptomatic rate accurately.

The asymptomatic rate can be estimated clinically (Johansson *et al.*, 2021; Martinelli *et al.*, 2022; Sah *et al.*, 2021); while this is the most accurate approach, it requires large-scale surveillance testing, which can be prohibitively resource-intensive. In contrast, computational approaches tend to add compartments to traditional SIR models, which is also our strategy. In Li *et al.* (2021), they add multiple compartments; we add just one asymptomatic compartment, which simplifies our model and increases computational tractability. Our approach is most similar to Layton and Sadria (2022), but we generalize to a larger geography and incorporate commuter data. For any modeling task, especially with a novel infection, aggregating different modeling approaches and data sources usually yields a strong consensus estimate; therefore, we hope to add to the literature by providing a modeling refinement and an additional estimate of the asymptomatic rate of spread to the ensemble.

## 2 Methods

### 2.1 The model

We show how to embed the classic SIR epidemiological model (Kermack and McKendrick, 1927) within a geographic network with known travel rates. The network G=(V,E) is a directed graph joining *N* nodes (typically, counties), whose edges are annotated with the corresponding mean traffic rates of commuters. The edge set *E* includes all the pairs (i,j) such that residents of county *i* commute to work in county *j*. We assume the availability of an *N*-by-*N* stochastic “commute” matrix *M*, such that $M_{ij}$ indicates the probability that someone commutes from county *i* to county *j* on a typical workday.

On day *t*, we denote the number of susceptible and recovered individuals in county *i* by $s_{i}(t)$ and $r_{i}(t)$, respectively. Among the $f_{i}(t)$ carriers of the virus in the county, we distinguish between the $c_{i}(t)$ of them who show symptoms and the $a_{i}(t)=f_{i}(t)-c_{i}(t)$ who do not. The population size in county *i* is denoted by $n_{i}=f_{i}(t)+s_{i}(t)+r_{i}(t)$ and is assumed fixed over the period under investigation. For convenience, we may write the right-hand side as $\sum_{x\in \{f,s,r\}}x_{i}(t)$.

The commute matrix *M* is blind to the health status of commuters. Symptomatic people tend to travel less, however, and this change has great effect on contagion. To capture this phenomenon, we introduce the *decommute rate* δ∈[0,1] as a measure of the propensity of people feeling sick to stay home:


(1) $$M^{c}=(1-\delta )M+\delta I.$$

where I represents the identity matrix. Note that, if δ=0, being symptomatic has no bearing on commuting. The matrix $M^{c}$ is a symmetry-breaking device which allows to distinguish between sick virus carriers and the rest. This difference creates observable nonlinearities in the viral dynamics that we can exploit to estimate the asymptomatic rate ρ. While our model could allow $M^{x}$ to be a function of time, we simply require $M^{c}$ to be different from the other transition matrices in order to get the benefit of symmetry-breaking. Therefore, we let all of them be static and set $M^{s}=M^{a}=M^{r}=M$. In the results reported here, we set δ to 8/9.

#### 2.1.1 The chronology of infection

*Instead of stating the model all at once, we introduce it one piece at a time, following its natural chronology. We fix a county i and trace the changes in the main state variables s, c, a, r, f, n. We use specific times for illustrative purposes only.*

- **Step 1:** At 8 am on day *t*, all commuters are ready to go to work. We have $f_{i}(t)=c_{i}(t)+a_{i}(t)$ and $\sum_{x\in \{s,c,a,r\}}x_{i}(t)=n_{i}(t)=n_{i}$.
- **Step 2:** At 9 am, commuters are at work. This changes the local population into a transient one, which we denote with a “hat.” By definition of the commute matrix, ${\hat{x}}_{i}(t)=\sum_{j}M_{ji}^{x}x_{j}(t)$ for x=s,c,a,r, with ${\hat{f}}_{i}(t)=\sum_{x\in \{c,a\}}{\hat{x}}_{j}(t)$ and ${\hat{n}}_{i}(t)=\sum_{x\in \{s,f,r\}}{\hat{x}}_{j}(t)$. The transient population at county *i* will now get to mix all day at work and spread the infection among itself.
- **Step 3:** At 5 pm, commuters go home. The new population at county *i* is denoted with a “bar.” It consists of the same $n_{i}$ people present at 8 am, but with a different health status distribution. Take the set of infected individuals: it includes the $f_{i}(t)$ carriers from 8 am plus the newly infected. The latter consist of the subset of the $s_{i}(t)$ susceptible individuals who caught the virus by commuting to county *j* and got exposed to a carrier in the transient population of *j*. Note that this includes the case j=i of non-commuters who were exposed to infected visitors. The chance of anyone getting sick in this fashion is $\varphi_{j}(t):=\beta {\hat{f}}_{j}(t)/{\hat{n}}_{j}(t)$, where 0<β<1 measures the transmission rate: it is the average number of contacts per person per day times the probability of transmission in a contact between an infected person and a susceptible one. (The model can easily accommodate a time-varying rate β. The reason for keeping it fixed is to decouple the baseline socialization rate from its pandemic-induced variations via the commute matrices.)
  The number of newly infected residents of county *i* is the sum, over all *j*, of the number of commuters from county *i* who went to county *j* and got infected there: therefore, it is equal to $s_{i}(t)\psi_{i}(t)$, where $\psi_{i}(t):=\sum_{j}M_{ij}\varphi_{j}(t)<1$ denotes the *worktime infectivity rate*: it is the probability that a commuter from *i* catches the virus at work.
  We have ${\bar{f}}_{i}(t)=f_{i}(t)+s_{i}(t)\psi_{i}(t)$. Since a fraction ρ of these new infections are asymptomatic, we have
  (2) $$\{\begin{array}{c} {\bar{s}}_{i}(t)=s_{i}(t)(1-\psi_{i}(t))\\ {\bar{c}}_{i}(t)=c_{i}(t)+(1-\rho )s_{i}(t)\psi_{i}(t)\\ {\bar{a}}_{i}(t)=a_{i}(t)+\rho s_{i}(t)\psi_{i}(t). \end{array}$$

- **Step 4:** At 8 am on day t+1, further mixing will have occurred in county *i* since the previous evening. A fraction γ of the infected people will have recovered by then. Writing

$$u_{i}(t)=\beta {\bar{s}}_{i}(t)\left(\frac{{\bar{c}}_{i}(t)+{\bar{a}}_{i}(t)}{n_{i}}\right),$$

we have


(3) $$\{\begin{array}{c} s_{i}(t+1)={\bar{s}}_{i}(t)-u_{i}(t)\\ c_{i}(t+1)=(1-\gamma ){\bar{c}}_{i}(t)+(1-\rho )u_{i}(t)\\ a_{i}(t+1)=(1-\gamma ){\bar{a}}_{i}(t)+\rho u_{i}(t)\\ r_{i}(t+1)=r_{i}(t)+\gamma {\bar{c}}_{i}(t)+\gamma \bar{a}(t). \end{array}$$

We note that traSIR involves two rounds of mixing: the first one in the daytime accounts for intercounty infection (via commuting); the second one (nighttime) models intracounty infection (within each county). For simplicity, we model recovery in the latter only. (For this reason, our value of γ might differ from the standard one by a factor of 2.)

#### 2.1.2 traSIR in vector form

We can give a compact description of the model. Let x(t) denote the row vector with *N* coordinates $x_{i}(t)$, for x∈{s,c,a,r,n}. We define the row vectors


(4) $$\{\begin{array}{c} \hat{x}(t)=x(t)M^{x}(t),\;\text{for}\;x\in \{s,c,a,r\}\\ \hat{f}(t)=\sum_{x\in \{c,a\}}\hat{x}(t)\;;\;\;\;\hat{n}(t)=\sum_{x\in \{s,f,r\}}\hat{x}(t)\\ \varphi (t)=\beta \hat{f}(t)⊘\hat{n}(t)\;;\;\;\;\psi_{t}=\varphi (t)M^{s}{\left(t\right)}^{T}. \end{array}$$

Using the symbols ⊗ and ⊘ to refer to component-wise vector multiplication and division, respectively, we have


(5) $$\{\begin{array}{c} \bar{s}(t)=s(t)-s(t)⊗\psi (t)\\ \bar{c}(t)=c(t)+(1-\rho )s(t)⊗\psi (t)\\ \bar{a}(t)=a(t)+\rho s(t)⊗\psi (t). \end{array}$$

For $u(t):=\beta \bar{s}(t)⊗(\bar{c}(t)+\bar{a}(t))⊘(n_{1},\ldots ,n_{N})$,


(6) $$\{\begin{array}{c} s(t+1)=\bar{s}(t)-u(t)\\ c(t+1)=(1-\gamma )\bar{c}(t)+(1-\rho )u(t)\\ a(t+1)=(1-\gamma )\bar{a}(t)+\rho u(t)\\ r(t+1)=r(t)+\gamma \bar{c}(t)+\gamma \bar{a}(t). \end{array}$$

### 2.2 Parameter estimation

Given a commute network and daily symptomatic infections across each node in the network, we develop an approach for estimating the asymptomatic rate ρ. The estimation algorithm can be viewed as a two-player game in which participants take turns updating their current estimates of (β,γ) and ρ, respectively. Recall that β,γ measure the infection and recovery rate, respectively. We assume that all counties have the same value of β and γ. The updating is driven by grid search (and gradient descent) with respect to a normalized mean-square loss function, which is computed for a node *k* across all time points as follows: where *c* is the vector in ${\left[0,1\right]}^{T}$ whose coordinate c(t) denotes the recorded rate of symptomatic cases in the population in some given county *k* at time *t*. We write $c=c_{k}$ when disambiguation is needed.


(7) $$L(c,\hat{c})=\sum_{t=1}^{T}{\left(\frac{c(t)}{||c|{|}_{\infty }}-\frac{\hat{c}(t)}{||\hat{c}|{|}_{\infty }}\right)}^{2},$$

The normalization makes the loss invariant under scaling. This is a necessary feature given the noise in the data. Of highest concern is the corruption of the official figures caused by the inclusion of reported asymptomatic cases via testing and the exclusion of symptomatic patients who do not seek a diagnosis. We assume that the signal-to-noise ratio remains constant over time; hence, that the time series *c* is available up to an unknown scaling factor. The normalization factors out that uncertainty.

The vector $\hat{c}=\hat{c}(\beta ,\gamma ,\rho )$ is the traSIR-predicted counterpart to the factual vector *c*; the matrix *M* and the decommute rate, defined in (1), are fixed. We assume that the infection is seeded at county $i_{0}$. With ρ expected to exert a relatively minor influence on the transmission/recovery parameters at the seeded node, it is natural to base the estimate of (β,γ) on the time series $c_{i_{0}}$.

Algorithm 1 procedure Estimate(*c*)

  $\rho^{*}\leftarrow 0.5$

  **for**$\ell =1,2,\ldots ,j_{\text{max}}$**do**

   ▹ use grid search to optimize $\left(\beta^{*},\gamma^{*}\right)$ via normalized mean-square loss function at initial county $i_{0}$

   $\left(\beta^{*},\gamma^{*})\leftarrow \arg\min_{\left(\beta ,\gamma \right)}L(c_{i_{0}},{\hat{c}}_{i_{0}}(\beta ,\gamma ,\rho^{*})\right)$

   ▹ use grid search to optimize $\rho^{*}$ via normalized mean-square loss function across all counties

   $\rho^{*}\leftarrow \arg\min_{\rho }\sum_{i=1}^{N}L(c_{i},{\hat{c}}_{i}(\beta^{*},\gamma^{*},\rho ))$

   ▹ gradient descent on $\rho^{*}$

   $g(x):=\sum_{i=1}^{N}L(c_{i},{\hat{c}}_{i}(\beta^{*},\gamma^{*},x))$

   k←0; τ←∞

   **while**$\tau >\tau_{\text{min}}\;\&\;k<k_{\text{max}}$**do**

    $\tau \leftarrow \varepsilon (dg/dx)(\rho^{*})$

    $\rho^{*}\leftarrow \max\{0,\rho^{*}-\tau \}$

    k←k+1

   **return**$\left(\beta^{*},\gamma^{*},\rho^{*}\right)$

Within Algorithm 1, we set $j_{\text{max}}=3$ (convergence is quick). The grid search is over a discrete space of size $10^{3}$ for ρ and $10^{4}$ for (β,γ). The number of gradient descent steps is $k_{\text{max}}=10^{3}$; the gradient descent threshold is $\tau_{\text{min}}=10^{-12}$ and the learning rate is $\varepsilon =10^{-4}/NT$. Note that the output $\left(\beta^{*},\gamma^{*},\rho^{*}\right)$ will be referred to as the estimated parameters $\left(\hat{\beta },\hat{\gamma },\hat{\rho }\right)$ in the text.

### 2.3 Actual data

**Figure 1.**
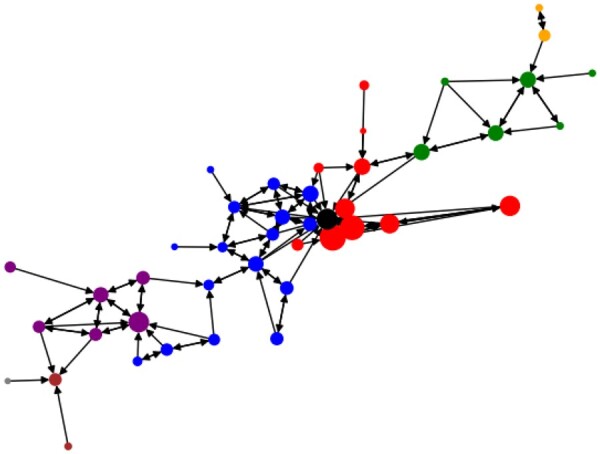
New York City metropolitan area: Each state is colored differently, with, at the center, Manhattan in black. The node size corresponds to the population in that county. The nodes are positioned according to the geographic center of each county

For the commute network and population data, we rely on the most recent (pre-COVID) *American Community Survey* from the U.S. Census Bureau (American Community Survey [ACS], 2015a,b). The nodes in the network represent the counties; the edges are directed and weighted in proportion to the number of residents who live in the source county and work in the destination county. We clean up the data by removing all the edges associated with fewer than 10 000 commuters. From the resulting graph, we extract the largest weakly connected component, which in this case corresponds to the New York Metropolitan Area. It consists of 44 counties: a visualization of which can be seen in Figure 1. For the infection data, we use the New York Times COVID-19 tracker and focus on the 200 days between March 1, 2020 and September 17, 2020 (Connell, 2022; The COVID Tracking Project at The Atlantic, 2022; The New York Times, 2022) (https://www.census.gov/data/tables/2015/demo/metro-micro/commuting-flows-2015.html and https://www.census.gov/programs-surveys/acs/technical-documentation/table-and-geography-changes/2015/5-year.html).

### 2.4 Simulated data

We generate 481 low-discrepancy values of β, γ and ρ, where 0.2≤β,ρ≤0.8,0.01≤γ≤0.7, using Sobol sequences from the SciPy package (https://docs.scipy.org/doc/scipy/reference/generated/scipy.stats.qmc.Sobol.html). For each of the 481 combinations of parameters, we run traSIR with the corresponding parameters for 150 timesteps on the New York Metropolitan area population and network data, assuming that there is a single infected individual in New York County (Manhattan). We further corrupt the resulting symptomatic population sizes by a fixed scalar that is unknown to the algorithm.

For validation, we run Algorithm 1 on the corrupted simulation to produce the estimated parameters $\left(\hat{\beta },\hat{\gamma },\hat{\rho }\right)$. We evaluate the accuracy and tabulate the residuals between the estimation and actual parameters (β,γ,ρ).

## 3 Results

The main contribution of this article is to demonstrate empirically that a network-based epidemiological model can uncover key parameters of a contagious disease. We provided an intuitive explanation for why this might be possible as long as a symmetry-breaking mechanism is in place for distinguishing among different types of virus carriers. Before we discuss the empirical evidence and validate our approach, we provide a succinct mathematical foundation for our claim.

### 3.1 Theoretical analysis

We fix the county *i* and the time *t* and we drop all mention of *t* when it is understood from the context. By Equations (2) and (3), where


(8) $$\begin{array}{c} f_{i}(t+1)=(1-\gamma +\beta {\bar{s}}_{i}/n_{i}){\bar{f}}_{i}\\ =(1-\gamma +\beta s_{i}(1-\psi_{i})/n_{i})(f_{i}+s_{i}\psi_{i})\\ =(1-\gamma +\beta s_{i}/n_{i})f_{i}\\ +(1-\gamma +\beta (s_{i}-f_{i})/n_{i})s_{i}\psi_{i}-(\beta /n_{i}){\left(s_{i}\psi_{i}\right)}^{2}, \end{array}$$

$$\begin{array}{c} \psi_{i}=\sum_{j}M_{ij}^{s}\varphi_{j}(t)=\beta \sum_{j}M_{ij}\frac{{\hat{f}}_{j}}{{\hat{n}}_{j}}=\beta \sum_{j}M_{ij}\frac{\sum_{k}\left(f_{k}-\delta c_{k}\right)M_{kj}+\delta c_{j}}{\sum_{k}\left(n_{k}-\delta c_{k}\right)M_{kj}+\delta c_{j}}. \end{array}$$

Recall that ${\hat{f}}_{i}(t)$ denotes the number of infected individuals in the transient population at county *i* at the end of the morning commute. Let $f_{i}^{\prime }=\sum_{k}f_{k}M_{ki}$ be the number it would have been if we had δ=0 and hence $M^{c}=M$; we derive $n_{i}^{\prime }=\sum_{k}n_{k}M_{ki}$ from ${\hat{n}}_{i}(t)$ likewise. We have where $g_{j}=f_{j}^{\prime }-f_{j}$. This allows us to rewrite $\psi_{i}$ as


(9) $$\{\begin{array}{c} {\hat{f}}_{j}(t)=\sum_{k}\left(f_{k}-\delta c_{k}\right)M_{kj}+\delta c_{j}=f_{j}^{\prime }-\delta (1-\rho )g_{j}\\ {\hat{n}}_{j}(t)=\sum_{k}\left(n_{k}-\delta c_{k}\right)M_{kj}+\delta c_{j}=n_{j}^{\prime }-\delta (1-\rho )g_{j}, \end{array}$$

(10) $$\psi_{i}=\beta \sum_{j}M_{ij}\left(\frac{f_{j}^{\prime }-\delta (1-\rho )g_{j}}{n_{j}^{\prime }-\delta (1-\rho )g_{j}}\right).$$

The worktime infectivity rate $\psi_{i}$ plays a key role in traSIR. If M=I, then $\psi_{i}=\beta f_{i}/n_{i}$ is the usual infectivity rate in the classic SIR model. Take the case of an arbitrary matrix *M* and set δ=0. Denote by $E_{j(i)}$ the expectation operator indexed by *i* and defined by $M_{ij}$, for j=1,…,N. Likewise, we introduce the expectation operator $E_{k(j)}$, indexed by *j* and defined by $n_{k}M_{kj}/\sum_{l}n_{l}M_{lj}$, for k=1,…,N. It follows that


(11) $$\begin{array}{c} \psi_{i}^{|\delta =0}=\beta \sum_{j}M_{ij}\sum_{k}\left(\frac{n_{k}M_{kj}}{\sum_{l}n_{l}M_{lj}}\right)\frac{f_{k}}{n_{k}}=\beta E_{j}E_{k(j)}\frac{f_{k}}{n_{k}}. \end{array}$$

We conclude that, when decommuting is withheld (δ=0), $\psi_{i}$ is an average of infection ratios $f_{k}/n_{k}$ over counties adjacent to *i* or adjacent to the latter. This two-degree of separation corresponds to individuals from distinct counties meeting at work in a third county. The same idea holds for δ>0, but with corrective terms that we discuss below.

In epidemiology, an important characteristic of an infection is the basic reproduction number $R_{0}$, which measures the average number of cases generated by an infected individual (Anderson and May, 1991). At the outset of the pandemic, we can use $f_{i}(t+1)/f_{i}(t)$ as a proxy for the reproduction number $R_{0}$ associated with county *i*. In a classical SIR model, $R_{0}$ has the form β/γ, but in TraSIR, it follows from (8) that


(12) $$\begin{array}{c} R_{0}=1-\gamma +\frac{\beta s_{i}}{n_{i}}+\left(\frac{1-\gamma +\beta (s_{i}-f_{i})/n_{i}}{f_{i}}\right)s_{i}\psi_{i}\\ -\frac{\beta }{f_{i}n_{i}}{\left(s_{i}\psi_{i}\right)}^{2}. \end{array}$$

Together, Equations (10) and (12) form a system S(ρ)=0, which in theory allows us to recover the asymptomatic rate ρ from β, γ and $R_{0}$. It is noteworthy that this requires decommuting. The system S cannot be solved for ρ in closed form. Using traSIR for estimation can thus be viewed as a numerical solver for S.

### 3.2 Simulations

We demonstrate that Algorithm 1 can accurately recover the infection rate β, recovery rate γ and asymptomatic rate ρ in simulated infections across a wide range of parameters, using just knowledge about the network and the numbers of symptomatic infected individuals. For each of simulations resulting from many combinations of parameters (see Methods), we will use the number of symptomatic individuals for each county over time. In practice, the actual number of symptomatic individuals is larger than the number reported, we multiply each of the resulting symptomatic population sizes by a fixed scalar (unknown to the algorithm), and then run Algorithm 1 to produce the estimated parameters $\left(\hat{\beta },\hat{\gamma },\hat{\rho }\right)$.

**Figure 2.**
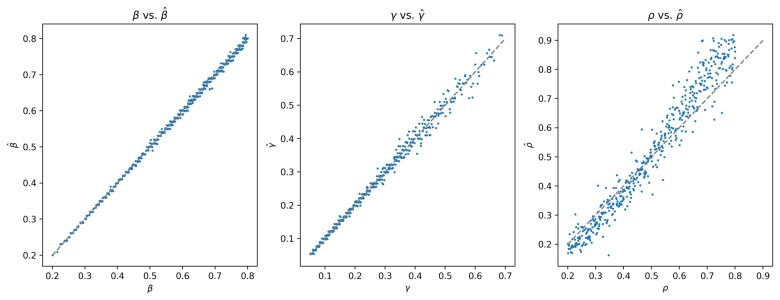
Each plot compares the actual and predicted value of a parameter for many different combinations of the two others. As expected, the estimation of ρ degrades as the actual value gets large. Ultimately, if no one feels sick, behavior does not change and the method cannot pick up ρ

We find excellent agreement between the actual parameters β, γ and ρ and their estimates $\left(\hat{\beta },\hat{\gamma },\hat{\rho }\right)$ (Fig. 2). Figure 2 shows a scatter plot of an estimated parameter against the corresponding synthetic parameter for the New York area. The Pearson correlation coefficient is 0.9996 between β and its predicted value. For γ and ρ, it is 0.9983 and 0.9915, respectively. The absolute residual across all starting parameters has mean 0.023 and standard deviation 0.017. The absolute residual mean and standard deviation for β are 0.0032 and 0.00246; for γ are 0.0065 and 0.0064 and for ρ are 0.0158 and 0.0163.

### 3.3 Applications to COVID-19 data

Having validated our estimation technique on simulated data, we now apply Algorithm 1 to daily infection numbers from the New York Metropolitan area (see Methods), and estimate the asymptomatic rate ρ, a parameter of critical importance to health policymakers. We find:


β=0.320   ;  γ=0.046   ;  ρ=0.345 .

**Figure 3.**
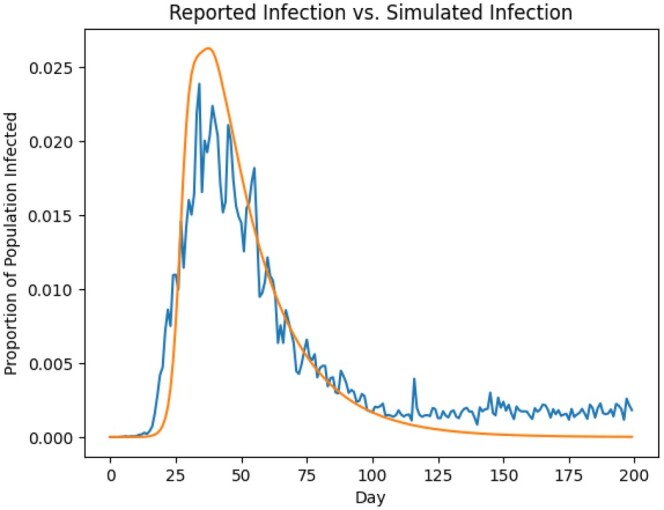
The blue ragged line is the scaled version of the reported daily case count across the New York City metro area, summed across the 44 counties considered here. The orange smooth line is the simulated daily symptomatic case count given our estimated parameters. The scaling factor is chosen to display both lines in a similar magnitude

Using these parameters, we also compared the traSIR-simulated symptomatic infection count with the real reported infection count across the New York metropolitan area (Fig. 3), and find good agreement.

## 4 Discussion

We have shown via theoretical analysis and simulation that a network-augmented compartmental model can effectively estimate the asymptomatic rate of viral infections using only data about symptomatic infections. The theoretical estimation is derived through (10) and (12). For simulation, we have applied this approach to actual COVID-19 data and derived an estimate of the asymptomatic rate that matches well with the latest estimates obtained via extensive random testing.

While our results are based on the different interaction patterns among asymptomatic and symptomatic populations, it is also possible to distinguish between these two types of spread in two more ways:

1. Distinct infection rates: we can impose dissimilar transmissibility rates in the two types of spread.
2. Differentiated seeding: we can account for several types of spread starting in various locations, perhaps based on local policy or other environmental factors.

Taken together, these factors are a broad set of levers that can influence the emergent behavior of our model, thus potentially enhancing the accuracy of our traSIR model.

Our estimates for β and γ are sharper than for ρ. This is no surprise. Both the transmission rate and the recovery rate have direct influence on the local shape of the infection time series: the first one has a large effect on the ascent phase of the contagion while the other one’s impact can be felt most acutely in the descent phase. The impact of the asymptomatic rate ρ is more global and subtle. It can be felt in the speed of the traveling waves and generally operates on longer time scales. TraSIR is able to leverage such information. Credit for our success must also go to sheer luck: An asymptomatic rate of ∼30% is almost ideally sized for estimation. As we observed earlier, a rate close to 100% would make the task hopelessly difficult. This leaves open the possibility that other nonlinearities in the system can be exploited to boost accuracy when needed. While fast-changing health policy measures and medical breakthroughs (e.g. vaccination) can present traSIR with major challenges, they also create new windows of opportunity for novel estimation mechanisms. We hope that this work will plant the seeds for exciting new research on the messy, difficult, but fascinating subject of uncovering hidden epidemiological parameters.
